# Dataset of transcriptome assembly of date palm embryogenic calli and functional annotation

**DOI:** 10.1016/j.dib.2020.105760

**Published:** 2020-05-25

**Authors:** S. Naganeeswaran, T.P. Fayas, M.K. Rajesh

**Affiliations:** ICAR-Central Plantation Crops Research Institute, Kasaragod 671124, Kerala, India

**Keywords:** Date palm, Somatic embryogenesis, Embryogenic calli, Transcriptome, Annotation

## Abstract

Date palm (*Phoenix dactylifera* L.; 2n = 36; Arecaceae), cultivated in tropical and sub-tropical regions worldwide, is a staple food for people in the Middle East region and has economic value throughout the world. Tissue culture is considered as a feasible technique for the large-scale multiplication of elite date palm varieties. In this article, we report the transcriptome assembly from the embryogenic calli of Khalas variety of date palm. A total of 50,852,331 paired-end (PE) raw reads were acquired using an Illumina sequencing platform. Reference-based assembly, with date palm genome, resulted in 53251 transcripts. A total of 63888 Gene Ontology (GO) terms could be annotated from the assembled transcriptome. Also, transcription factor families and small RNAs were annotated from the assembled transcriptome. Results of the pathway analysis revealed that a total of 2584 transcripts were involved in various metabolic pathways. Transcripts with possible roles in somatic embryogenesis were also identified. The dataset provides insights into the expression pattern of various genes during early somatic embryogenesis in date palm.

Specifications table

**Table:** 

Subject	Agriculture and Biological Sciences
Specific subject area	Plant transcriptomics
Type of data	Tables, figure, text file
How data were acquired	Illumina HiSeq TM 2000 sequencing platform
Data format	Raw, filtered, analyzed
Parameters for data collection	Embryogenic calli were generated from juvenile leaf explants of Khalas variety of date palm on Murashige and Skoog (MS) medium [1] supplemented with 2,4-D (100 mg/L), 2iP (3 mg/L) and sucrose (60 g/L). Three-month old friable embryogenic calli were used for total RNA extraction, cDNA library preparation and sequencing.
Description of data collection	The RNA-seq dataset was collected from paired-end sequencing of date palm embryogenic calli cDNA library using Illumina HiSeq 2000^TM^ platform. The raw reads were recorded in a FASTQ file. Raw reads were filtered to remove reads containing adapter or reads of low quality, and clean reads were mapped to reference date palm genome. Gene expression estimation and annotation were then carried out.
Data source location	Kasaragod, India (12°32′38.0"N; 74°57′45.7"E).
Data accessibility	Repository name: NCBI SRA
	Data identification number: PRJNA238431
	Direct URL to data: https://www.ncbi.nlm.nih.gov/sra/SRX474412.

## Value of the data

• This dataset allows the discovery of genes which are differentially expressed during early somatic embryogenesis in date palm.
• The RNA-seq dataset would enable identification of marker genes indicating the transition of somatic cells to embryogenic cells and aid researchers working on date palm tissue culture in early identification of embryogenic calli.
• This knowledge gained will also enable understanding of the molecular mechanisms that underlie the intricate regulatory networks regulating date palm somatic embryogenesis.
• It would also enable the characterization of genes and the corresponding proteins that are conserved during somatic embryogenesis in various palms.

## 1 Data Description

**Fig. 1:**
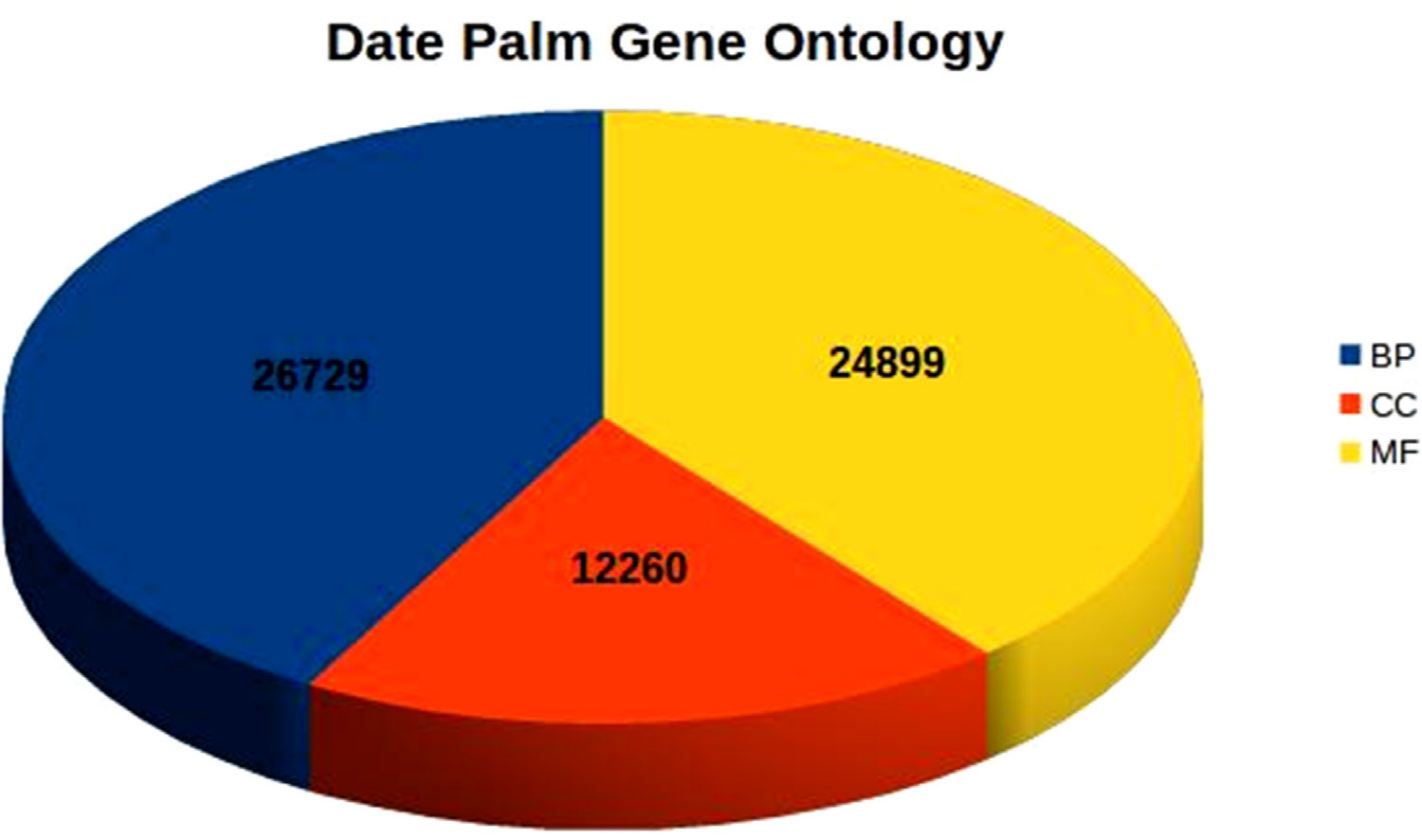
Annotated Gene Ontology (GO) terms in date palm embryogenic calli transcriptome (BP: Biological Process; CC: Cellular Component; MF: Molecular Function).

Details of raw reads generated, assembly and annotation information are provided in Table 1. Supplementary table S1 provides an overview of FPKM gene expression estimation for each transcript. Annotated Gene Ontology (GO) terms in date palm embryogenic calli transcriptome are given in Fig. 1 and Supplementary table S2. The details of transcription factors and small RNAs, annotated from the assembled transcriptome, are provided in Supplementary tables S3 and S4 respectively. The results of the pathway analysis of transcripts involved in various metabolic pathways are given in Supplementary table S5. Transcripts with possible roles in date palm somatic embryogenesis are given in Supplementary table S6.

**Table 1:** Details of raw reads generated, assembly and annotation information.

Total raw reads	50,852,331
Mapped reads	43,987,268 (∼87%)
Total transcripts generated	53,251
G+C percentage	45.1
Annotated transcripts using the Uniprot protein database	39,968
Number of transcripts involved in pathways	2,584
Number of GO terms identified	63,888

A total of 50,852,331 paired-end (PE) reads were generated for the date palm embryogenic calli transcriptome. Alignment of good quality reads against the date palm reference genome resulted in ∼87% of read alignment (43,987,268 reads) and 53,251 transcripts (Table 1). Similarity search against Uniprot plant protein database resulted in 75% of assembled transcripts showing significant similarity with plant database (Table 1). A total of 63,888 GO terms (Fig. 1) were annotated from the assembled transcriptome (Supplementary table S2). Transcription factor families annotated from the assembled transcriptome include bHLH, MYB, WRKY, bZIP, ERF, C2H2, NAC etc. (Supplementary table S3). A total of 122 small RNAs could also be annotated (Supplementary table S4). Results of the pathway analysis revealed that 2,584 transcripts were involved in various metabolic pathways (Supplementary table S5). The enriched KEGG pathways were dominantly represented by “metabolic pathways” including carbohydrate, energy, lipid, nucleic acid and amino acid metabolism. The transcripts possessing possible roles in date palm somatic embryogenesis include APETALA2 (AP2) class of transcription factors, auxin responsive factors (ARFs), leafy cotyledon (LEC), late embryogenesis-abundant protein (LEA), mitogen-activated protein kinase (MAPK), somatic embryogenesis receptor kinase (SERK), small auxin up RNA-like (SAUR-like) auxin-responsive family, WRKY and WUSCHEL (WUS) transcription factors (Supplementary table S6).

## 2 Experimental Design, Materials, and Methods

### 2.1 Experimental design and sampling

Full strength MS medium, supplemented with 60 g/L sucrose with 1 mg/L GA_3,_ was utilized for germination of fresh, mature seeds of Khalas cultivar of date palm. Juvenile leaf explants of zygotic embryo-derived plantlets were used as explants for callogenesis. Callus induction was achieved in MS medium supplemented with 2,4-D (100 mg/L), 2iP (3 mg/L) and sucrose (60 g/L). The *in vitro* cultures were maintained in the dark at 27 ± 2^o^ C for callus initiation. Callus induction was observed within two weeks of culturing. Sub-culturing was undertaken at 60-day intervals under the same culture conditions. Friable embryogenic calli were developed within three months of culture initiation and these calli were sampled for transcriptome analysis.

### 2.2 RNA extraction and sequencing

A total of five biological replicates (100 mg each) were pooled together for isolation of RNA. RNA isolation was performed using Trizol reagent (SIGMA) and treated with DNase I (Fermentas, USA) according to the manufacturer's instructions. Illumina compatible NGS library preparation were performed using the method described by Rajesh et al. [2]. Transcriptome sequencing was carried out using Illumina HiSeq2000 platform [100 bp paired-end (PE) chemistry]. Adapter contamination and low-quality regions (Q < 20) towards 3’ end were trimmed out using Cutadapt program [3]. The final quality of processed reads was accessed using FastQC tool [4].

### 2.3 Data analysis

Transcriptome alignment, assembly to reference genome [5] and gene expression estimation was carried out via Tophat [6] and Cufflinks [7] tools.

### 2.4 Transcriptome functional annotation

The BLAST similarity analysis was performed using the assembled transcriptome against Uniprot Viridiplantae database [8], Plant transcription factor database [9] and small RNA database [10]. Gene Ontology terms associated with the transcripts were extracted from the Uniprot database and integrated with the BLAST search results using an in-house Perl script. Metabolic pathway genes expressed in the transcriptome were identified by KAAS server [11]. In addition, the presence of genes known to be involved in somatic embryogenesis was identified as described in Rajesh et al. [2].

## Declaration of Competing Interest

The authors declare that they have no known competing financial interests or personal relationships that could have appeared to influence the work reported in this paper.

## Appendix Supplementary materials

Image, application 1

Image, application 2

Image, application 3

Image, application 4

Image, application 5

Image, application 6

Image, application 7

Image, application 8
