# Supplementary material for: Dataset of transcriptome assembly of date palm embryogenic calli and functional annotation
Source: Data Brief. 2020 May 25;31:105760. doi: 10.1016/j.dib.2020.105760 (PMC7270187; doi:10.1016/j.dib.2020.105760)
Supplement: Supplementary file 1 [file mmc1.docx]

**Supplementary Tables**

Table S1: Transcript FPKM values of date palm embryogenic calli transcriptome; Table S2: BLAST similarity search against Uniprot plant database; Table S3: Similarity search against Plant Transcription Factor database; Table S4: Similarity search against small RNA database; Table S5: Expressed KEGG pathway genes; Supplementary table S6: Transcripts related to somatic embryogenesis.
